# 

**Published:** 1856-07

**Authors:** 


### CONTENTS.

ORIGINAL COMMUNICATIONS— PAGE.
Dysenteria Intermittent. By Henry W. Davis, M. D., Paris, Ill., ........ 295
Laceration of the Cornea of Both Eyes, during a Convulsion. Reported by J. W. McKinney, M.D., of New Albany, Ill., ........ 305
Case of Pleuro-Tuberculosis. By F. B. Norcom, M.D., Chicago, Ill., May, 1856 ........ 307
BOOK NOTICES, ........ 312
MEDICAL SOCIETIES—
Sixth Annual Meeting of the Illinois State Medical Society, ........ 318
Semi-Annual Meeting of the Æsculapian Society, ........ 327
Annual Meeting of the Henry County Medical Society, ........ 334
EDITORIAL, ........ 335

